# Associations between disordered gut microbiota and changes of neurotransmitters and short-chain fatty acids in depressed mice

**DOI:** 10.1038/s41398-020-01038-3

**Published:** 2020-10-16

**Authors:** Min Wu, Tian Tian, Qiang Mao, Tao Zou, Chan-juan Zhou, Jing Xie, Jian-jun Chen

**Affiliations:** 1 Deparment of Neurosurgery, Guizhou Provincial People’s Hospital, 550000 Guiyang, Guizhou Province China; 2 Department of Neurology, Affiliated Hospital of Guizhou Medical University, 550004 Guiyang, Guizhou Province China; 3 Department of Pharmacy, The Second Affiliated Hospital of Chongqing Medical University, 400010 Chongqing, China; 4 Department of Psychiatry, Affiliated Hospital of Guizhou Medical University, 550004 Guiyang, Guizhou Province China; 5 NHC Key Laboratory of Diagnosis and Treatment on Brain Functional Diseases, Chongqing Medical University, 400016 Chongqing, China; 6 Chongqing Emergency Medical Center, Department of Endocrinology and Nephrology, the Fourth People’s Hospital of Chongqing, Central Hospital of Chongqing University, 400014 Chongqing, China; 7 Institute of Life Sciences, Chongqing Medical University, 400016 Chongqing, China

**Keywords:** Molecular neuroscience, Depression

## Abstract

Mounting evidence suggests that gut microbiota can play an important role in pathophysiology of depression, but its specific molecular mechanisms are still unclear. This study was conducted to explore the associations between changes in neurotransmitters and short-chain fatty acids (SCFAs) and altered gut microbiota in depressed mice. Here, the chronic restraint stress (CRS) model of depression was built. The classical behavioral tests were conducted to assess the depressive-like behaviors of mice. The 16S rRNA gene sequence extracted from fecal samples was used to assess the gut microbial composition. Liquid and gas chromatography mass spectroscopy were used to identify neurotransmitters in hypothalamus and SCFAs in fecal samples, respectively. Finally, 29 differential bacteria taxa between depressed mice and control mice were identified, and the most differentially abundant bacteria taxa were genus Allobaculum and family Ruminococcaceae between the two groups. The acetic acid, propionic acid, pentanoic acid, norepinephrine, 5-HIAA and 5-HT were significantly decreased in depressed mice compared to control mice. Genus Allobaculum was found to be significantly positively correlated with acetic acid and 5-HT. Taken together, these results provided novel microbial and metabolic frameworks for understanding the role of microbiota-gut-brain axis in depression, and suggested new insights to pave the way for novel therapeutic methods.

## Introduction

Depression is a debilitating disease that involves a loss of interest in activities, a low mood and a persistent feeling of sadness. Nowadays, depression has become the leading cause of disability globally and affects nearly 6% of the adult population worldwide each year^1^. It is the result of complex gene-environment interactions. The genetic contribution to this disease is expected to be nearly 35%, and environmental factors (such as sexual and stress) are also strongly related with the development of depression^2,3^. Many theories have been developed to explain the pathogenesis of depression, such neurotransmission deficiency and endocrine-immune system dysfunction^4,5^. But none of these theories has been universally accepted. Thus, it is urgently needed to find a novel pathophysiologic mechanisms underlying depression.

Currently, there is growing evidence indicating that gut microbiota can play an important role in the pathophysiology of depression^6–8^. Zheng et al. reported that gut microbiota might be involved in the onset of depressive-like behaviors by regulating gut-brain axis glycerophospholipid metabolism^6^. Another study showed that gut microbiota could increase the depression-like behavior and inflammatory processes in the ventral hippocampus of stress vulnerable individuals^7^. Our studies in humans have observed differences in gut microbiota composition between patients with depression and healthy controls^9–11^. Other studies also found the altered gut microbiota composition in depressed patients^12,13^. These studies have successfully identified variations in some bacteria taxa, such as *phyla* Bacteroidetes, *phyla* Actinobacteria and *genera* Alistipes. Meanwhile, we have pioneered the fecal transplantation model of depression by transferring the feces from patients with depression to germ-free mice to induce depressive-like behavior^9^. These preliminary results suggested the close relationship between depression and disordered gut microbiota, but could not show a causal relationship between the differential gut microbial compositions and depression. Nowadays, there has been limited direct evidence linking depression and gut microbiota, and no causal connection has yet been proven.

Emerging evidence also indicates that gut microbiota can affect the host brain function through the microbiota-gut-brain (MGB) axis^14,15^. But the specific signaling mechanisms in the MGB axis are still unclear. Gut microbiota can produce many substances, such as short-chain fatty acids (SCFAs), neurotransmitters and gut hormones. Neurotransmitters, such as norepinephrine, dopamine and 5-hydroxytryptamine (5-HT), can be synthesized by gut microbiota^16^. In some cases, gut microbiota can regulate the 5-HT signaling by mediating the tryptophan metabolism^17^. SCFAs, such as acetic acid, propionic acid, and butyric acid, can enter the circulatory system; then it is possible that gut microbiota may signal to host brain via this route^18^. Therefore, in order to explore the possible mechanisms involved in the crosstalk between brain function and gut microbiota, we conducted this study to investigate the associations between altered gut microbiota and changes of neurotransmitters in hypothalamus and SCFAs in fecal samples in depressed mice.

Chronic stress plays a critical role in the development of depression. McEwen et al. reported that repeat stress, such as 21 days of CRS, could result in structural and functional changes in brain regions^19^. Some brain regions, such as prefrontal cortex and hypothalamus, are associated with cognition, emotion regulation and learning^20^. Currently, chronic stress, including CRS and chronic mild stress (CMS), is the most widely used animal model of depression^21^. But, two drawbacks limit the widespread use of CMS model: difficult to replicate across laboratories and high operation costs along with the long period^22^. In contrast, CRS has been widely used in stress-induced depression model because of its low operation costs and readily accessible stress operation procedure^23^. Therefore, we used CRS-induced depression model in this study.

## Methods and materials

### Male adult C57BL/6 mice

Male adult C57BL/6 mice (8–16 weeks of age; about 20 g) were purchased from the Laboratory Animal Center of Chongqing Medical University and used to build CRS model of depression. The mice were individually housed under standard conditions: 12 h light/dark cycle; 22 ± 1 °C temperature; 52 ± 2% humidity; free access to food and water. This study was approved by the Ethics Committee of Chongqing Medical University (Approval No. 20170301). The whole procedures were conducted according to the National Institutes of Health Guidelines for Animal Research (Guide for the Care and Use of Laboratory Animals, NIH Publication No.8023, revised 1996). When the experiment began, the mice were randomly assigned into two groups using a computer generated randomization table. In one group, mice were exposed to CRS by placing them in 50ml-plastic tubes. There were a few holes on the tubes to keep air flow for 4 h per day. During the restraint time, the mice were under water and food deprivation. In another group, mice were free access to food and water without restraint. The total process continued 28 consecutive days. To ensure that the possible differences between the two groups could be detected, the number of mice was set to 20 in each group. Meanwhile, ten mice in each group were randomly selected to measure gut microbiota composition, neurotransmitters and SCFAs. In many previous studies, the number of mice was set to eight in each group to assess the differences on metabolite, protein or gut microbiota composition between the two groups^24,25^. Thus, the sample sizes in this study were likely to result in sufficient power, even if power analysis was not performed.

### Behaviors testing

Open field test (OFT): each mouse was individually put at the center of box (45 × 45 × 45 cm). To remove olfactory cues, the 70% alcohol and water were used to clean the box after each test. The mice were allowed to explore the box for 6 min. The total distance, center distance and center time in the last 5 min were recorded; Forced swim test (FST): the mice were assigned to do a pre-test (15 min) swim one day before FST. Each mouse was individually put in a Plexigals cylinder (15 cm diameter and 30 cm height) filled with 18 cm of water (24 ± 1 °C). The water was replaced after each test. The immobility time was recorded in the 5 min period; Sucrose preference test (SPT): the mice were trained to adapt to a 1% sucrose solution 72 h before SPT: two bottles with 1% sucrose solution were placed, and 24 h later, the liquid in one bottle was replaced with water for 24 h. In the last 24 h period, the mice were under water and food deprivation. After adaption, the mice were free access to two bottles: one containing water and the other one containing 1% sucrose solution. The positions of the two bottles (left or right sides) were randomly changed. The liquid consumptions in 24 h were recorded, and the sucrose preference was defined as [100% × sucrose consumption/(sucrose consumption + water consumption). The investigator was blinded to the group allocation and outcomes assessment.

### Neurotransmitter identification

First, we added 200 μl ultrapure water (1% formic acid, FA) into the hypothalamus tissue, and the mixture were homogenized for three times in a disposable glass tube. Then, 800 μl (1% FA) pre-cooling pure acetonitrile was added into the mixture for vortex mixing. Later, we put the homogenate in the ice bath (−20 °C) for 1 h incubation, and the obtained protein deposition were induced in an ice bath for 20 min by ultrasound. Fourthly, the samples were centrifuged at 14,000 × *g* × 20 min under 4 °C, and the obtained supernatant was transferred into a glass vial to conduct vacuum-drying. When conducting mass spectrometry detection, 100 μl (1% FA) ACN/water (1:1, v/v) were added into the samples to dissolve. Then, the mixture was centrifuged at 14,000 × *g* × 20 min under 4 °C. Finally, the obtained supernatant was used to identify neurotransmitters using LC-MS system. The detailed information of LC-MS procedure was displayed in Supplementary file 1.

### SCFAs identification

First, sample preparation was conducted: (1) added 1 ml NaOH solution (5 mmol/l) into 50 mg fecal samples (pre-cooled under 4 °C); (2) the mixture was homogenized for 3 min (sample tray was pre-cooled at −20 degrees), and then ultrasonically extracted for 7 min in an ice bath; (3) after centrifugation (12,000 r.p.m. × 10 min, 4 °C), transferred 500 μl supernatant into an injection vial and then added 300 μl pure water. Then, sample derivation was conducted: (1) added 500 μl propanol/pyridine (3:2, v/v) and 100 μl propyl chloroformate into the injection vial, conducted vortex for 10 s and then sonicate for 1 min; (2) added 300 μl n-hexane into the injection vial and conducted vortex (2000 r.m.p. × 60 s); (3) after centrifugation (12,000 r.p.m. × 5 min, 4 °C), transferred 250 μl obtained n-hexane layer into a new injection vial; (4) continually added 200 μl n-hexane into the original injection vial and conducted vortex (2000 r.m.p. × 60 s); (5) after centrifugation (12,000 r.p.m. × 5 min, 4 °C), transferred 200 μl n-hexane layer into the new injection vial; (6) added 10 mg anhydrous sodium sulfate into the obtained 450 μl n-hexane layer and conducted vortex for 10 s, and then analyzed using GC-MS system. Later, standards derivation was conducted: (1) added 300 μl mixed standards solution and 500 μl NaOH solution (0.005 mol/l) into an injection vial; (2) added 500 μl propanol/pyridine (3:2, v/v) and 100 μl propyl chloroformate into the injection vial, conducted vortex for 10 s and then sonicate for 1 min; (3) added 300 μl n-hexane into the injection vial and conducted vortex (2000 rmp × 60 s); (4) after centrifugation (12,000 r.p.m. × 5 min, 4 °C), transferred 250 μl obtained n-hexane layer into a new injection vial; (5) continually added 200 μl n-hexane into the original injection vial and conducted vortex (2000 r.m.p. × 60 s); (6) after centrifugation (12,000 r.p.m. × 5 min, 4 °C), transferred 200 μl n-hexane layer into the new injection vial; (7) added 10 mg anhydrous sodium sulfate into the obtained 450 μl n-hexane layer and conducted vortex for 10 s, and then analyzed using GC-MS system. The detailed information of GC-MS procedure was displayed in Supplementary file 1.

### 16S rRNA gene sequencing

Fecal samples were collected from the mice, and then immediately frozen and stored under −80 °C before analysis. We used the mortar and pestle to pulverize the fecal samples, and used the standard power soil kit protocol to extract the bacterial genomic DNA. Briefly, the fecal samples were thawed on the ice. Then, we added the MoBio lysis buffer into the fecal samples and conducted vortex mixing. The obtained fecal suspensions were centrifuged. Finally, we put the obtained supernatant into the MoBio Garnet bead tubes containing MoBio buffer. We used the Roche 454 sequencing system to extract the V3-V5 regions of 16s rRNA gene sequences from the fecal samples, and the obtained gene sequences were PCR-amplified with barcoded universal primers.

We used Mothur (Version 1.31.2, http://www.mothur.org/) to quality-filter the obtained raw gene sequences to collect unique reads. Sequences with <200 bp or >1000 bp, as well as sequences with any barcode mismatches, ambiguous bases, homopolymer runs exceeding six bases and primer mismatches were excluded. The remained sequences were assigned to operational taxonomic units (OTUs) with >=97% pairwise sequence identity. The Ribosomal Database Project (RDP) reference database was used to taxonomically classify the obtained OTUs.

### Statistical analysis

The independent samples *t*-test (if the data fit the normal distribution), nonparametric Mann–Whitney U test (if the data do not fit the normal distribution), pearson correlation analysis or spearman correlation analysis was conducted when appropriate. The variances between the two groups were calculated using Levene’s test; if the variances are not similar between the two groups, the adjusted *p*-value was used. Meanwhile, we constructed the summaries of the taxonomic distributions of OTUs to calculate the relative abundances of gut microbiota at different levels. Four different parameters (shannon, simpson, phylogenetic diversity and chao) were used to assess the alpha diversity. Distance matrices (beta diversity) between samples were assessed using principal coordinate analysis (PCoA). Random Forest algorithm was conducted to find the key discriminatory OTUs. The linear discriminant-analysis effect size (LEfSe) was further used to identify the dominant bacteria taxa in both control mice and depressed mice. *P*-value <0.05 was considered significant. The method of Benjamini and Hochberg False Discovery was used to perform multiple testing corrections.

## Results

### Mice exposed to CRS showed depressive-like and anxiety-like behaviors

**Fig. 1:**
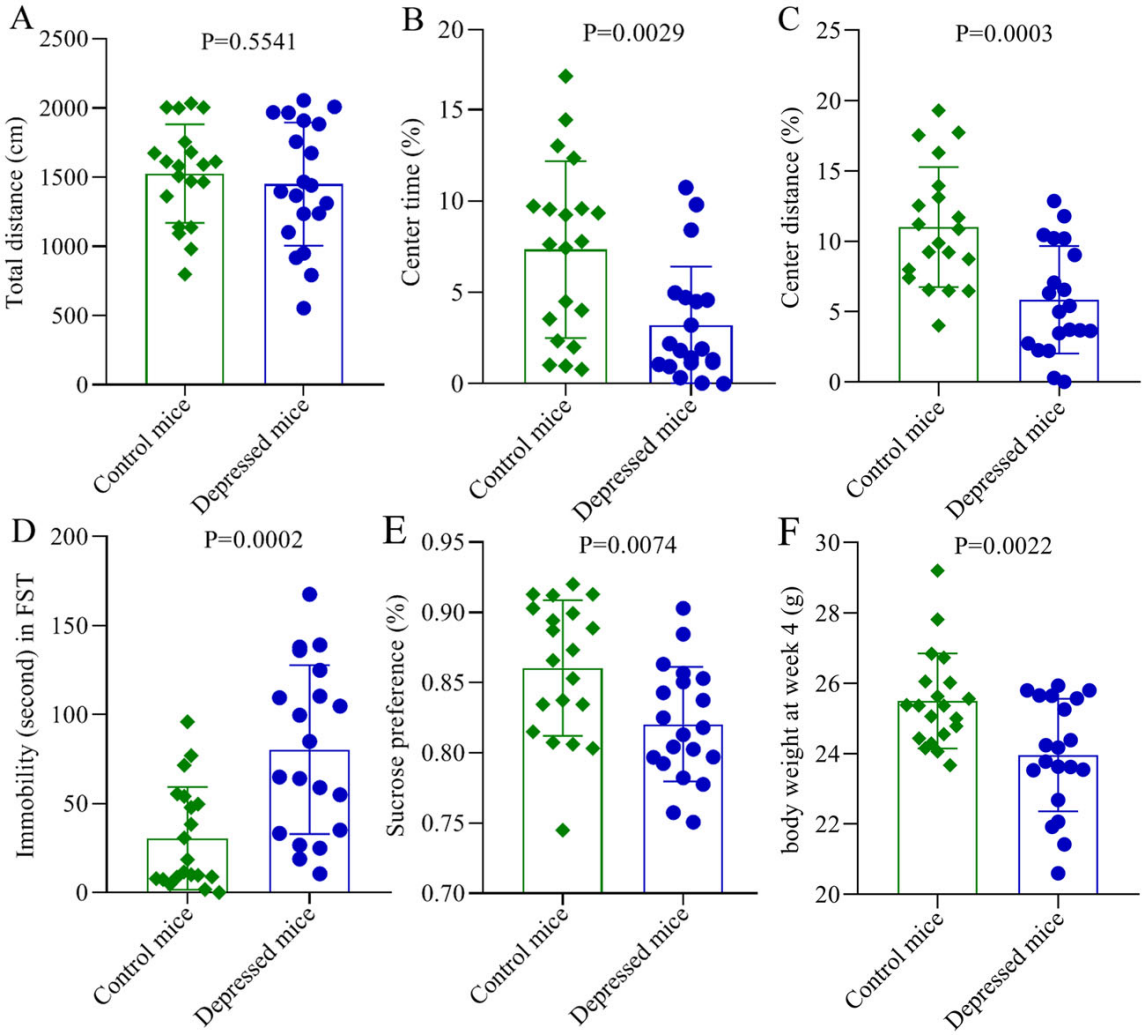
Chronic restraint stress-induced depressive-like and anxiety-like behaviors in mice. **A** No significant difference was found in total distance between the two groups in OFT; (**B**, **C**) the depressed mice had significantly decreased center time (**B**) and center distance (**C**) in OFT; (**D**) the depressed mice had significantly increased immobility time in FST; (**D**) the sucrose preference was significantly decreased in depressed mice in SPT. Twenty mice were in each group, and the error bars represent standard deviation.

The results of OFT (*n* = 20, each group) showed that there was no significant difference in total distance between control mice and depressed mice (*p* = 0.5541, Fig. 1A). In contrast, there was significant difference in center time (*p* = 0.0029, Fig. 1B) and center distance (*p* = 0.0003, Fig. 1C), suggesting the anxiety-like behavior in depressed mice. In the FST (*n* = 20, each group), immobility time was widely used as an index of depression-like behavior in the literature. Here, we found that the depressed mice had significantly increased immobility time compared to control mice (*p* = 0.0002, Fig. 1D). Meanwhile, the SPT (*n* = 20, each group) was used to assess anhedonia, which was also an index of depressive-like behavior. In this study, no significance was observed in sucrose preference prior to the CRS procedure. But, the sucrose preference was found to be significantly decreased in depressed mice compared to control mice at the end of the CRS procedure (*p* = 0.0074, Fig. 1E). In addition, no significant difference in body weight was found at baseline, but the body weight was significantly higher in control mice than in depressed mice at last (*p* = 0.0022, Fig. 1F). These results showed that the mice exposed to CRS showed depressive-like and anxiety-like behaviors.

### Disordered gut microbiota between control mice and depressed mice

**Fig. 2:**
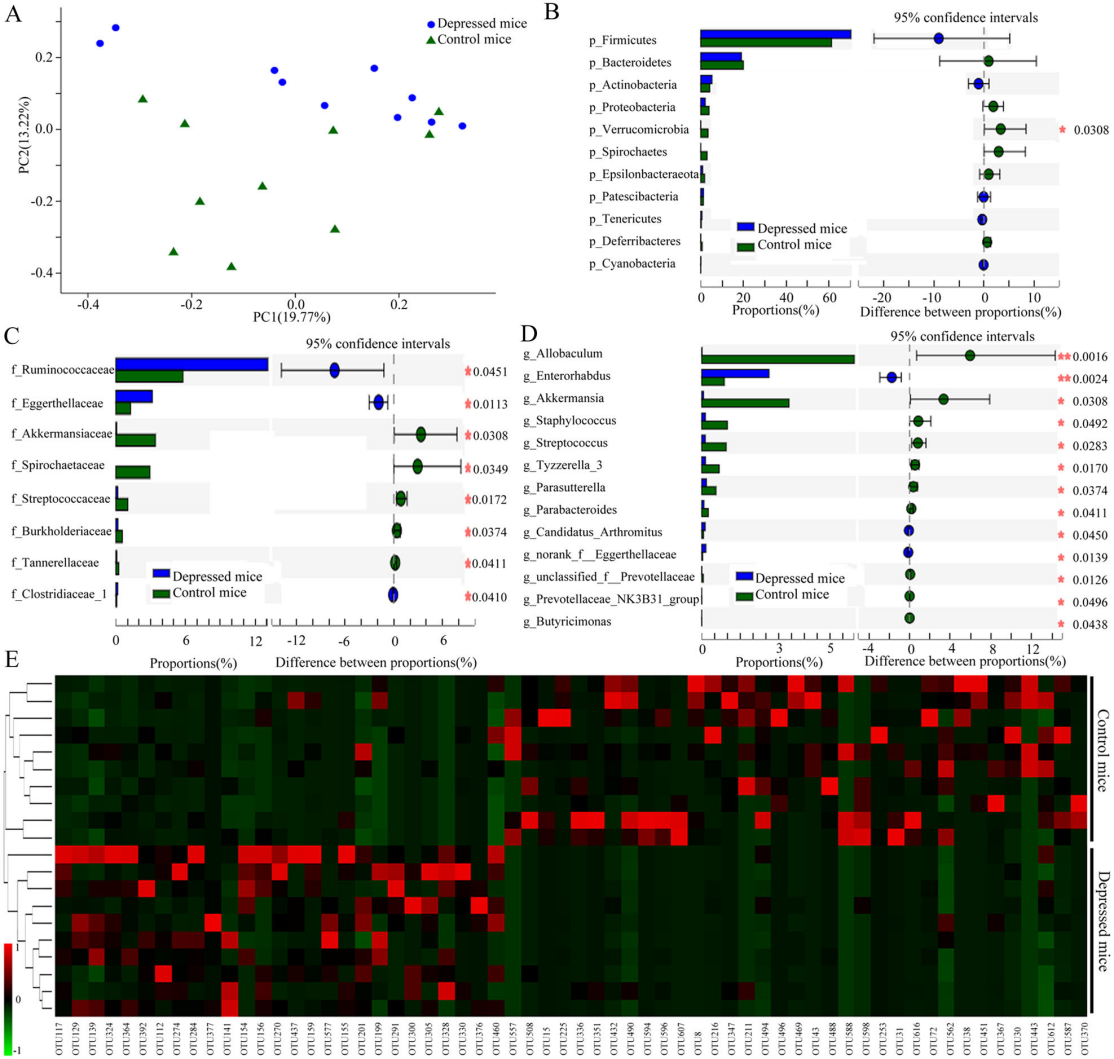
Significant alterations in gut microbiota in depressed mice compared to control mice. **A** PCoA showed an obvious difference in gut microbiotic composition between the two groups; (**B**) *phyla* Verrucomicrobia was significantly changed; (**C**) eight differential bacteria taxa on family level was identified; (**D**) 13 differential bacteria taxa on genus level were identified; (**E**) 62 OTUs key discriminatory OTUs whose relative abundance reliably distinguished control mice and depressed mice were identified. Ten mice were in each group.

The results of these four parameters showed that there was no significant difference in alpha diversity between control mice and depressed mice (*n* = 10/10, random samples) (shannon, *p* = 0.6394; simpson, *p* = 0.3468; phylogenetic diversity, *p* = 0.7862; chao, *p* = 0.6521). However, the results of PCoA showed the significant difference in the gut microbial community compositions between control mice and depressed mice (Fig. 2A). Further analysis found that *phyla* Verrucomicrobia (Fig. 2B), eight differential bacteria taxa on family level (Fig. 2C), and 13 differential bacteria taxa on genus level (Fig. 2D) were significantly changed in depressed mice. To find out the key discriminatory OTUs, Random Forest algorithm was used here. A total of 62 differential OTUs between control mice and depressed mice were identified (Fig. 2E). Of these differential OTUs, a total of 27 OTUs and 35 OTUs were overrepresented in depressed mice and control mice, respectively. The differential bacteria taxa on other levels between the two groups were displayed in Supplementary Fig. S1.

**Fig. 3:**
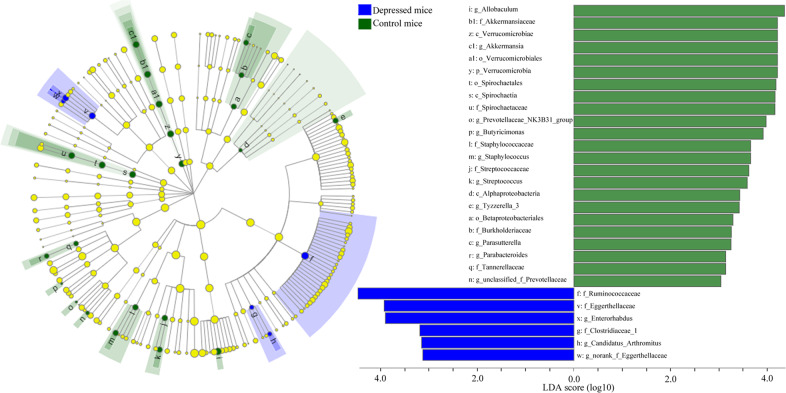
Dominant bacteria taxa in different group were identified using LEfSe. In total, 29 bacteria taxa with statistically significant and biologically consistent differences were found. The most differentially abundant bacteria taxa in control mice and depressed mice belonged to genus Allobaculum and family Ruminococcaceae, respectively. Ten mice were in each group.

To find out the dominant bacteria taxa in different groups, LEfSe was used in this study. It was a new method to identify the metagenomic biomarker by way of class comparison. In total, 29 bacteria taxa with statistically significant and biologically consistent differences were found here (Fig. 3). These bacteria taxa were the key phylotypes responsible for the different gut microbiota between control mice and depressed mice. The 23 bacteria taxa and 6 bacteria taxa were most abundant in control mice and depressed mice, respectively. The most differentially abundant bacteria taxa in control mice and depressed mice belonged to genus Allobaculum and family Ruminococcaceae, respectively.

Phylogenetic investigation of communities by reconstruction of unobserved states (PICRSt) was a common approach to conduct functional prediction of 16S data using a database of reference genomes and marker gene data. In this study, we used this method to conduct the functional prediction of gut microbiota. Finally, we found that the Lipid transport and metabolism of gut microbiota was significantly affected in depressed mice (*p* = 0.0142); more specifically, Fatty acid degradation (*p* = 0.0033), Glycerophospholipid metabolism (*p* = 0.0226), Biosynthesis of unsaturated fatty acids (*p* = 0.04185), Synthesis and degradation of ketone bodies (*p* = 0.02624), and Glycerolipid metabolism (*p* = 0.03558) were significantly affected.

### Differentially expressed SCFAs levels in depressed mice

**Fig. 4:**
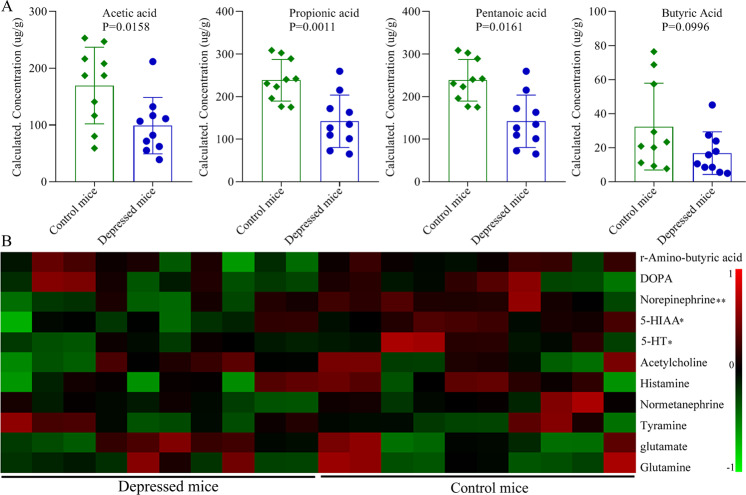
Differential SCFAs and neurotransmitters identification. **A** four kinds of SCFAs were detected, and acetic acid, propionic acid and pentanoic acid were found to be significantly decreased in depressed mice; (**B**) 11 kinds of neurotransmitters were detected, and norepinephrine, 5-HIAA and 5-HT were found to be significantly decreased in depressed mice. Ten mice were in each group, the error bars represent standard deviation, and two asterisks represents *p*-value < 0.01 and one asterisk represents *p*-value < 0.05.

SCFAs, as a major class of key bacterial metabolites, were very important for human health. In this study, seven major SCFAs (acetic acid, propionic acid, butyric acid, pentanoic acid, hexanoic acid, isobutyric acid and isovaleric acid) were measured in fecal sample of control mice and depressed (*n* = 10/10, random samples). But at last, only four major SCFAs (acetic acid, propionic acid, butyric acid, pentanoic acid) were successfully identified. The results showed that the levels of acetic acid (*p* = 0.0158), propionic acid (*p* = 0.0011) and pentanoic acid (*p* = 0.0161) were found to be significantly decreased in depressed mice compared to control mice (Fig. 4A); the level of butyric acid was not significantly different between the two groups (*p* = 0.0996) (Fig. 4A).

### Differentially expressed neurotransmitter levels in depressed mice

The perturbations in central and peripheral neurotransmitters were closely related with the pathogenesis of depression. In this study, ten kinds of neurotransmitters [r-Amino-butyric acid, dopamine, norepinephrine, 5-hydroxyindoleacetic acid (5-HIAA), 5-hydroxytryptamine (5HT), acetylcholine, histamine, tyramine, normetanephrine, glutamate and glutamine] were identified in control mice and depressed mice (*n* = 10/10, random samples). We obtained their relative concentration from the mass peak area of the sample analyte. The results showed that three kinds of neurotransmitters were significantly changed between the two groups. The levels of norepinephrine (*P* = 0.0081), 5-HIAA (*P* = 0.0133) and 5-HT (*P* = 0.0300) were found to be significantly decreased in depressed mice compared to control mice (Fig. 4B).

### Correlations between the differential SCFAs, neurotransmitters and bacteria taxa

**Fig. 5:**
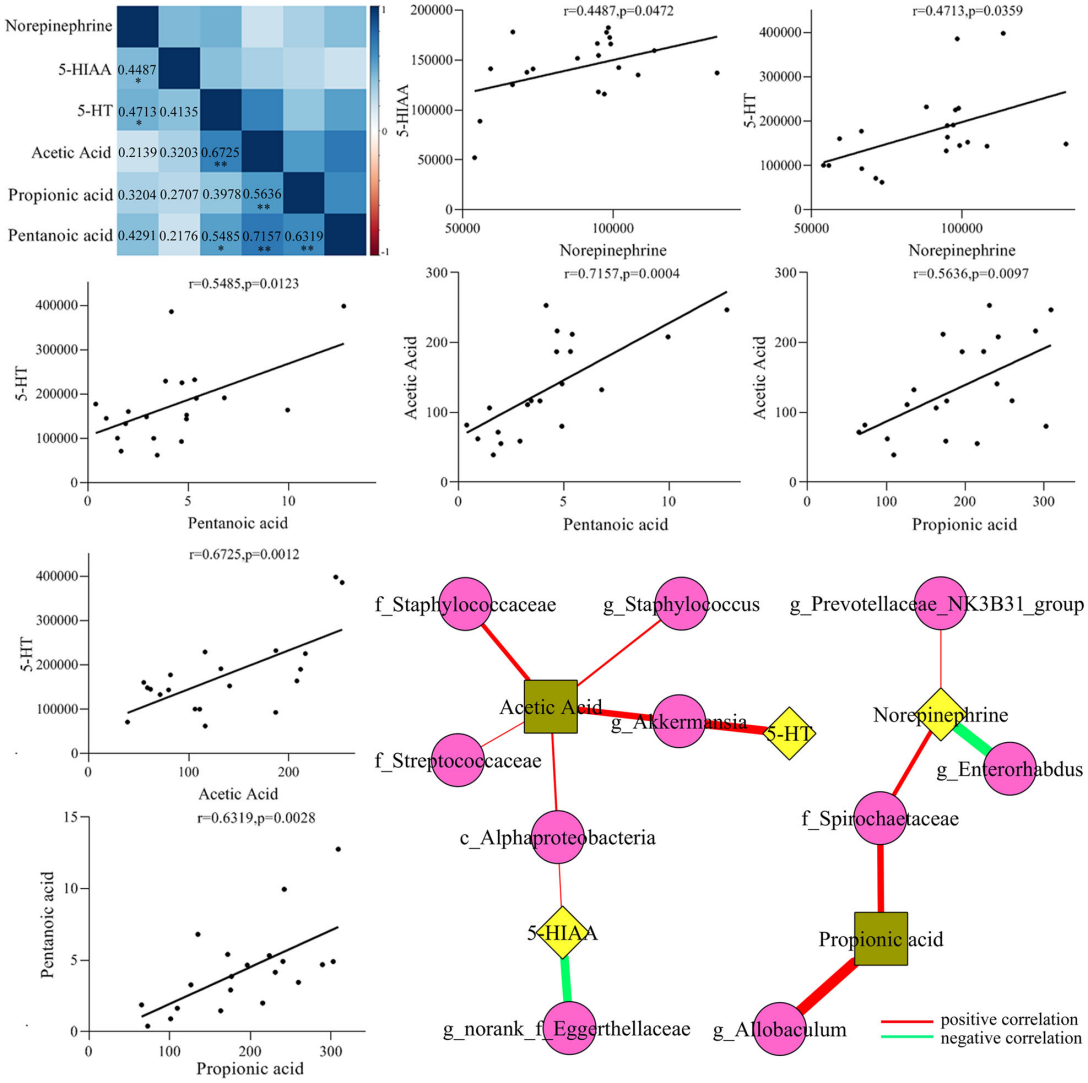
Correlations between the differential SCFAs, neurotransmitters and bacteria taxa. The top left corner was the heatmap of correlations between differential SCFAs and neurotransmitters. The bottom right corner was the network map of correlations between the differential SCFAs, neurotransmitters and bacteria taxa. The scatter plots were used to intuitively display the relationships between differential SCFAs and neurotransmitters.

In this study, we found that (Fig. 5): (i) propionic acid (*r* = 0.5636, *p* = 0.0097) and entanoic acid (*r* = 0.7157, *p* = 0.0004) were significantly positively correlated with acetic acid; (ii) there was significantly positive correlation between pentanoic acid and propionic acid (*r* = 0.6319, *p* = 0.0028); (iii) 5-HIAA (*r* = 0.4487, *p* = 0.0472) and 5-HT (*r* = 0.4713, *p* = 0.0359) were significantly positively correlated with norepinephrine; (iv) acetic acid (*r* = 0.6725, *p* = 0.0012) and pentanoic acid (*r* = 0.5485, *p* = 0.0123) were significantly positively correlated with 5-HT.

Meanwhile, we found that (Fig. 5): (i) there were significantly positive correlations between norepinephrine and two differential bacteria taxa: family Spirochaetaceae (*r* = 0.5490, *p* = 0.0122) and genus revotellaceae_NK3B31_group (*r* = 0.5110, *p* = 0.0212), and significantly negative correlation between norepinephrine and genus Enterorhabdus (*r* = −0.4631, *p* = 0.0396); (ii) 5-HIAA was significantly positively and negatively, respectively, correlated with class Alphaproteobacteria (*r* = 0.5141, *p* = 0.0204) and genus norank_f_Eggerthellaceae (*r* = −0.4600, *p* = 0.0413); (iii) a significantly positive correlation was found between genus Akkermansia and 5-HT (*r* = 0.5580, *p* = 0.0105); (iv) there were significantly positive correlations between acetic acid and five differential bacteria taxa: class Alphaproteobacteria (*r* = 0.5371, *p* = 0.0145), family Streptococcaceae (*r* = 0.5132, *p* = 0.0207), genus Staphylococcus (*r* = 0.4911, *p* = 0.0278), family Staphylococcaceae (*r* = 0.4892, *p* = 0.0287) and genus Akkermansia (*r* = 0.4450, *p* = 0.0495); and (v) both genus Allobaculum (*r* = 0.5991, *p* = 0.0052) and family Spirochaetaceae (*r* = 0.5490, *p* = 0.0122) were significantly positively correlated with propionic acid. Meanwhile, we did correlation analysis between all detected SCFAs, all detected neurotransmitters and differential bacteria taxa; the results were displayed in Supplementary Fig. S2.

## Discussion

Previous studies have been reported that chronic stress could result in the disturbance of gut microbiota and host metabolism, which might contribute to the development of depression^26,27^. Here, we found that the gut microbiota, SCFAs in fecal sample and neurotransmitters in hypothalamus were significantly changed in depressed mice compared to control mice. There were 23 bacteria taxa and 6 bacteria taxa significantly decreased and increased, respectively, in depressed mice. Three major SCFAs (acetic acid, propionic acid, and pentanoic acid) and three kinds of neurotransmitters (norepinephrine, 5-HIAA and 5-HT) were found to be significantly decreased in depressed mice compared to control mice. Meanwhile, the significantly correlations between differential bacteria taxa, SCFAs and neurotransmitters were found in this study. Our results indicated that gut microbiota might play an important role in the pathogenesis of depression by regulating the levels of SCFAs in fecal sample and neurotransmitters in hypothalamus.

Although many meaningful work have been done to expand our knowledge on the relationship between host and their gut microbiota, the underpinnings of MGB crosstalk is still unclear. As the key bacterial metabolites produced in the colon by gut microbiota, SCFAs are considered to have a critical role in neuro-immunoendocrine regulation. Meanwhile, it can directly regulate the size and function of the colonic Treg pool to remain colonic homeostasis and health^28^. The major products from microbial fermentative activity in the intestinal tract are SCFAs, in particular, acetic acid, butyric acid, and propionic acid^29^. In this study, we found that there were significantly positive correlations between some bacteria taxa with decreased relative abundances and two major SCFAs with decreased levels (acetic acid and propionic acid) in depressed mice. Previous studies also reported that the levels of SCFAs were decreased in naturally occurring depressive model of macaques and depressed patients^30,31^. Thus, considering the anti-inflammatory property of SCFAs, these differential bacteria taxa might be closely related to the development of depression by playing a role in the inflammation process.

Much evidence suggests that hypothalamic-pituitary-adrenal (HPA) axis is closely related with the pathophysiology of depression^32^. The dysregulation of HPA axis is found to be a common feature in patients with depression^33^. In this study, we found the significantly decreased levels of norepinephrine, 5-HIAA and 5-HT in the hypothalamus of depressed mice. Both 5-HT and norepinephrine are monoamine neurotransmitters that are closely associated with depression and widely accepted as target by most of antidepressants. Previous studies reported that gut microbiota could synthesize and release many neurotransmitters, such as norepinephrine and 5-HT^34,35^. Here, we found that some differential bacteria taxa were significantly related with 5-HT and norepinephrine in depressed mice. These results further demonstrated that besides regulating inflammatory response, gut microbiota could affect host brain function through the regulation of HPA axis.

Genus Akkermansia is one kind of probiotics in the intestinal tract. Bárcena et al. reported that transplantation with Akkermansia muciniphila was sufficient to exert beneficial effects for the healthspan and lifespan of mice^36^. In this study, we found the significantly decreased abundance of genus Akkermansia in depressed mice, and the significantly positive correlation between genus Akkermansia and 5-HT in hypothalamus. Some studies reported that more than 90% 5-HT might be compartmentalized in the gut^37,38^. Meanwhile, we also found the significantly positive correlation between genus Akkermansia and acetic acid. Interesting, as the most abundant SCFA, acetic acid can be produced from pyruvic acid via acetyl-CoA or Wood–Ljungdahl pathway by most enteric bacteria, such as genus Akkermansia^39^. These results indicated that genus Akkermansia might be a potential novel target for treating depression.

In many ways, SCFAs represent the signature hormones of gut microbiota. They may act as a bridge between gut microbiota and many functions assigned to them through classical endocrine signaling. For example, SCFAs can modulate the enteroendocrine 5-HT secretion^40^, an important neurotransmitter at multiple levels of MGB axis. Consistent with these findings, we found the significantly positive correlations between 5-HT in hypothalamus and two major SCFAs in fecal sample (acetic acid and pentanoic acid). As the most abundant SCFA in peripheral circulation, acetic acid can cross blood-brain barrier, and reduce appetite by activating the hypothalamic neurons driving satiety^41^. The interaction between host brain functions and gut microbiota is a complex relationship. Our findings demonstrated that SCFAs could be the agent of gut microbiota in affecting host brain functions.

In conclusion, we found that chronic stress could result in the gut microbiota dysbiosis: 29 differential bacteria taxa between depressed mice and control mice were identified. We also found that three major SCFAs in fecal sample and three kinds of neurotransmitters in hypothalamus were significantly decreased in depressed mice compared to control mice. Meanwhile, some significant correlations between differential bacteria taxa, SCFAs and neurotransmitters were identified. Our findings might suggest a possible pathway for depression: chronic stress disordered the gut microbiota, and the gut microbiota dysbiosis resulted in the changes of neurotransmitters in hypothalamus by regulating the levels of SCFAs in intestinal tract.

## Supplementary information

Supplemental File 1
