# Supplementary material for: Associations between disordered gut microbiota and changes of neurotransmitters and short-chain fatty acids in depressed mice
Source: Transl Psychiatry. 2020 Oct 16;10:350. doi: 10.1038/s41398-020-01038-3 (PMC7567879; doi:10.1038/s41398-020-01038-3)
Supplement: Supplementary file 1 — Supplemental File 1 [file 41398_2020_1038_MOESM1_ESM.doc]

**Supplementary information**

**Supplementary Figure S1 Differential bacteria taxa on class, order and species levels between the two group**


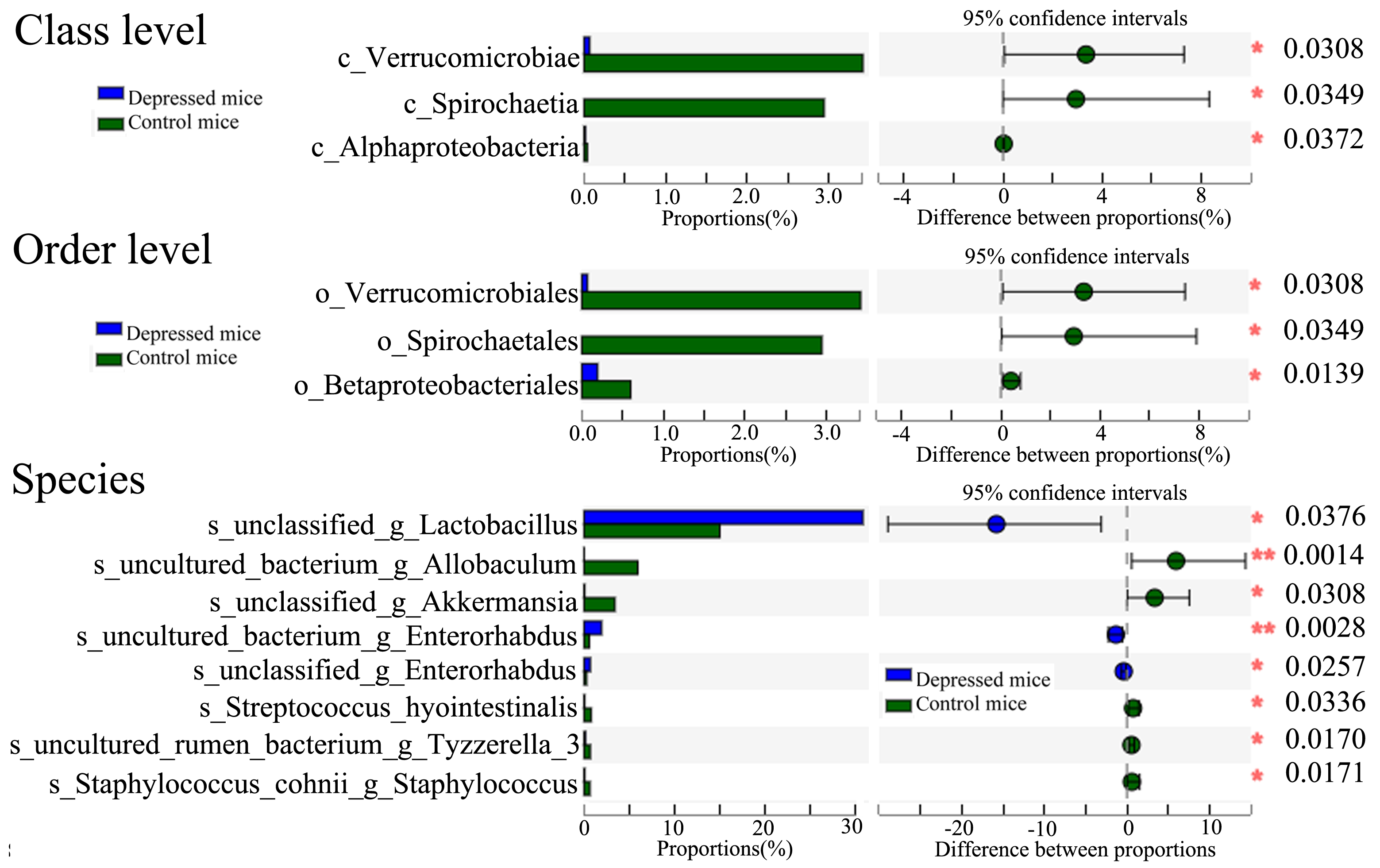


**Supplementary Fgiure S2** **Correlations between differential bacteria taxa, all detected SCFAs, all detected neurotransmitters.** DOPA, dopamine; 5-hydroxytryptamine, 5HT); 5-hydroxyindoleacetic acid, 5-HIAA.

**
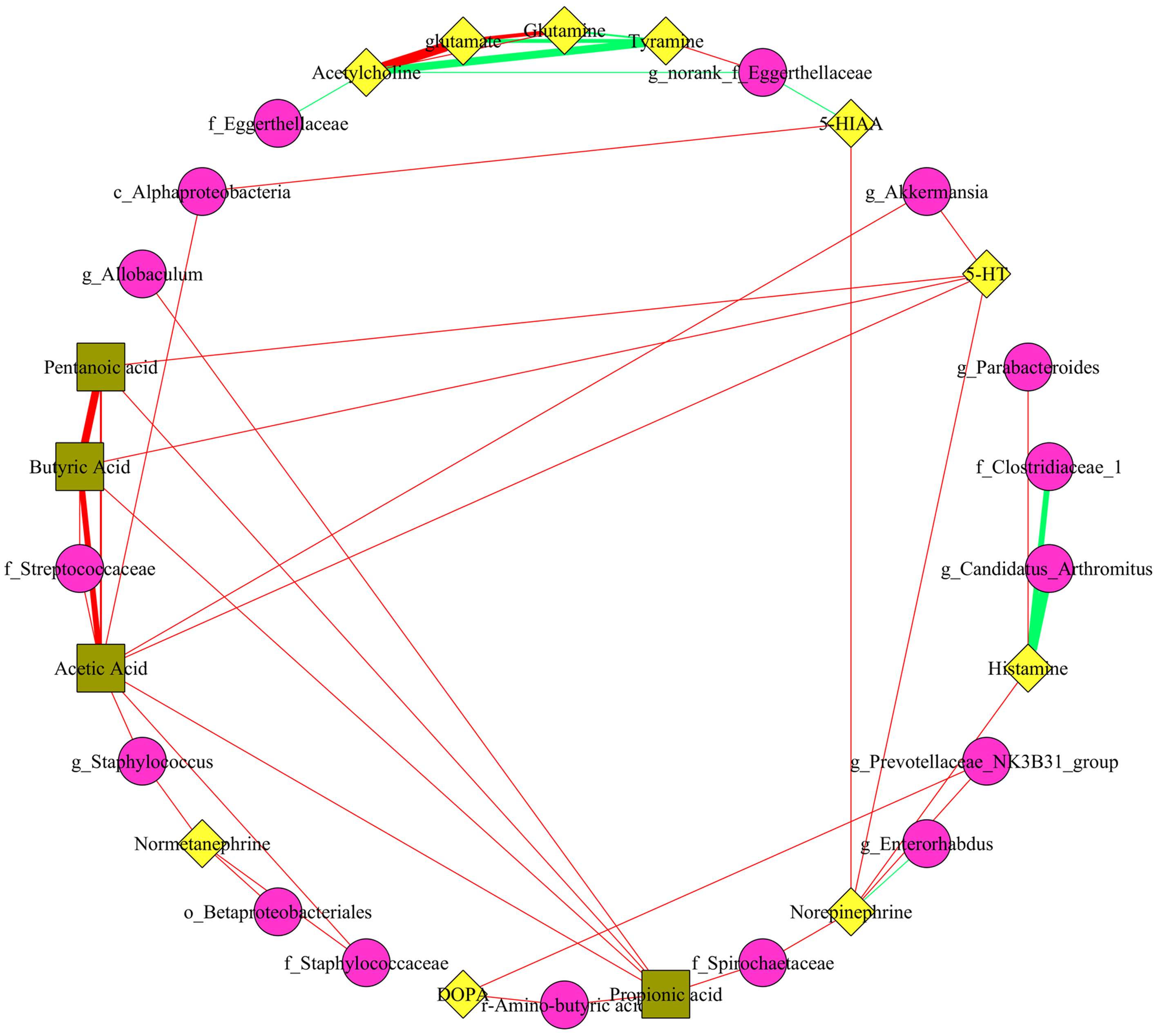
**

**Supplementary Methods**

**1. LC-MS procedure**

An auto-sampler Agilent 1290 HPLC system was used to separate the smaples. Two kinds of liquid were in the mobile phase: an aqueous solution containing 25 mM ammonium formate (0.1% FA) as liquid A; acetonitrile (0.1% FA) as liquid B. The 2μl samples were then put in 4°C automatic sampler (45°C ,column temperature; 300 μl/minute, flow velocity). The relevant liquid phase gradient: liquid B changes linearly from 90% to 40% during the first 18 minutes; then liquid B changes linearly from 40% to 90% using six seconds; at last, liquid B was maintained at 90% until to 24th mintue. To evaluate the repeatability and stability of system, quality control sample was set at specific intervals in the sample queue. Glutamate and gamma-aminobutyric acid were applied as standard for chromatographic retention time correction. The 5500 QTRAP mass spectrometer (AB SCIEX) was applied to conduct mass spectrometry analysis in positive ion mode. The 5500 QTRAP ESI source conditions: ion Spray Voltage Floating (ISVF), 5000 V; ion source gas1 (Gas1), 60; ion source gas2 (Gas2), 60; curtain gas (CUR), 30; source temperature, 450 °C. We used the MRM mode to detect neurotransmitters. The Multi-Quant software was used to extract the peak area and retention time of the chromatogram, and the corrected neurotransmitter retention time was applied to identify metabolites.

**2. GC-MS procedure**

A 5977B GC/MSD (with CTC automatic sampler) was used here. Chromatographic conditions: HP-5MS capillary column (30 m × 0.25mm × 0.25 μm, Agilent J&W Scientific, Folsom, CA, USA); high-purity helium (purity not less than 99.999%) as carrier gas; flow velocity, 1.0 mL/minute; and temperature of the injection port, 260°C; 1μl sample, split injection, split ratio 10:1, solvent delay 2.2minutes. Temperature programming: i) set the initial temperature of the column oven 50°C, and maintain for 5.2 minutes; ii) increase the temperature to 70°C with 10°C/minute, and maintain for 1.3 minutes; iii) increase the temperature to 85°C with 3°C/minute, and maintain for 1 minute; iv) increase the temperature to 110°C with 5°C/minute, and maintain for 1 minute; v) increase the temperature to 290°C with 30°C/minute, and maintain for 9 minute. Mass spectrometry conditions: electron bombardment ion source, ion source temperature of 230°C, quadrupole temperature of 150°C, electron energy of 70 eV. Scanning mode was full scan mode (SCAN), and quality scan range was set to: 30-600 m/z.

**3. Differential bacteria taxa on other levels**

As shown in Supplementary Figure S1, there were three differential bacteria taxa on class level (Verrucomicrobiae, Spirochaetia and Alphaproteobacteria) between the two groups; on order level, three differential bacteria taxa (Verrucomicrobiales, Spirochaetales and Betaproteobacteriales) were identified in depressed mice. Meanwhile, 25 differential bacteria taxa on species level were also found (only eight differential bacteria taxa on species level were displayed in Supplementary Figure S1). They are as following:

s_unclassified_g_Lactobacillus, s_uncultured_bacterium_g_Allobaculum, s_unclassified_g_Akkermansia, s_uncultured_bacterium_g_Enterorhabdus, s_unclassified_g_Enterorhabdus, s_Streptococcus_hyointestinalis, s_uncultured_rumen_bacterium_g_Tyzzerella_3, s_Staphylococcus_cohnii_g_Staphylococcus, s_uncultured_rumen_bacterium_g_Ruminococcaceae_UCG-014, s_uncultured_organism_g_Parasutterella, s_Desulfovibrio_sp._UNSW3caefatS, s_Candidatus_Arthromitus_sp._SFB-mouse-Japan, s_uncultured_Coriobacteriales_bacterium_g_norank_f_Eggerthellaceae, s_unclassified_g_Streptococcus, s_unclassified_g_norank_o_Mollicutes_RF39, s_unclassified_f_Prevotellaceae, s_uncultured_Firmicutes_bacterium_g_norank_o_Mollicutes_RF39

s_uncultured_Paenibacillaceae_bacterium_g_norank, s_Jeotgalicoccus_sp._M3T9B12, s_uncultured_bacterium_g_Prevotellaceae_NK3B31_group, s_Butyricimonas_virosa, s_uncultured_bacterium_g_norank_f_Coriobacteriales_Incertae_Sedis, s_uncultured_bacterium_g_Eubacterium]_coprostanoligenes_group, s_uncultured_rumen_bacterium_g_Ruminococcaceae_NK4A214_group and s_unclassified_g_Lactococcus

**4. Correlations between all detected SCFAs, neurotransmitters and differetial bacteria taxa**

Correlation analysis between all detected SCFAs, neurotransmitters and differential bacteria taxa was conducted. As shown in Supplementary Figure S2, there were significant correlations between some non-significantly changed neurotransmitters and some differential bacteria taxa, such as the significantly positive correlation between Histamine and genus Parabacteroides. Meanwhile, we found that there was significantly positive correlation between non-significantly changed Butyric Acid and family Streptococcaceae.
